# MR Scanner Systems Should Be Adequately Characterized in Diffusion-MRI of the Breast

**DOI:** 10.1371/journal.pone.0086280

**Published:** 2014-01-28

**Authors:** Marco Giannelli, Roberto Sghedoni, Chiara Iacconi, Mauro Iori, Antonio Claudio Traino, Maria Guerrisi, Mario Mascalchi, Nicola Toschi, Stefano Diciotti

**Affiliations:** 1 Medical Physics Unit, Pisa University Hospital “Azienda Ospedaliero-Universitaria Pisana”, Pisa, Italy; 2 Department of Oncology and Advanced Techniques, Medical Physics Unit, IRCCS-Arcispedale Santa Maria Nuova, Reggio Emilia, Italy; 3 Division of Radiology, Breast Unit, Massa Hospital, Azienda USL Massa e Carrara, Massa, Italy; 4 Department of Biomedicine and Prevention, Medical Physics Section, University of Rome “Tor Vergata”, Rome, Italy; 5 Department of Clinical and Experimental Biomedical Sciences, University of Florence, Florence, Italy; 6 Department of Radiology, Athinoula A. Martinos Center for Biomedical Imaging, Boston, Massachusetts, United States of America; 7 Harvard Medical School, Boston, Massachusetts, United States of America; 8 Department of Electrical, Electronic, and Information Engineering “Guglielmo Marconi”, University of Bologna, Cesena, Italy; Northwestern University Feinberg School of Medicine, United States of America

## Abstract

Breast imaging represents a relatively recent and promising field of application of quantitative diffusion-MRI techniques. In view of the importance of guaranteeing and assessing its reliability in clinical as well as research settings, the aim of this study was to specifically characterize how the main MR scanner system-related factors affect quantitative measurements in diffusion-MRI of the breast. In particular, phantom acquisitions were performed on three 1.5 T MR scanner systems by different manufacturers, all equipped with a dedicated multi-channel breast coil as well as acquisition sequences for diffusion-MRI of the breast. We assessed the accuracy, inter-scan and inter-scanner reproducibility of the mean apparent diffusion coefficient measured along the main orthogonal directions (<ADC>) as well as of diffusion-tensor imaging (DTI)-derived mean diffusivity (MD) measurements. Additionally, we estimated spatial non-uniformity of <ADC> (NU_<ADC>_) and MD (NU_MD_) maps. We showed that the signal-to-noise ratio as well as overall calibration of high strength diffusion gradients system in typical acquisition sequences for diffusion-MRI of the breast varied across MR scanner systems, introducing systematic bias in the measurements of diffusion indices. While <ADC> and MD values were not appreciably different from each other, they substantially varied across MR scanner systems. The mean of the accuracies of measured <ADC> and MD was in the range [−2.3%,11.9%], and the mean of the coefficients of variation for <ADC> and MD measurements across MR scanner systems was 6.8%. The coefficient of variation for repeated measurements of both <ADC> and MD was < 1%, while NU_<ADC>_ and NU_MD_ values were <4%. Our results highlight that MR scanner system-related factors can substantially affect quantitative diffusion-MRI of the breast. Therefore, a specific quality control program for assessing and monitoring the performance of MR scanner systems for diffusion-MRI of the breast is highly recommended at every site, especially in multicenter and longitudinal studies.

## Introduction

In magnetic resonance imaging (MRI), “diffusion” (i.e. the random, thermally-induced displacements of water molecules over time) [1] represents an extraordinarily sensitive contrast mechanism, and the exquisite structural detail it affords has proven useful in a vast number of clinical as well as research applications, especially in neuroimaging [2]. Currently, diffusion-MRI is a promising and potentially useful MRI technique for improving the diagnostic accuracy of breast imaging without administering contrast agents [3]–[8]. Indeed, previous studies have shown a potential role of quantitative diffusion-MRI in differentiating between benign and malignant breast lesions [9]–[12], with the majority of malignant lesions showing reduced diffusion when compared to benign lesions, and diffusion-MRI may aid in identifying patients with low grade ductal carcinoma in situ (DCIS) as compared to high grade DCIS, hence contributing to risk-stratification in DCIS [13], [14]. Some studies have revealed an inverse correlation between the cellularity of breast cancer and diffusion indices [15], [16], and diffusion indices have been seen to vary significantly according to various histopathological and immunohistochemical tumour features [17]. In locally advanced breast cancer, another potential application of diffusion-MRI is in the evaluation and assessment of the early response of cancer to neoadjuvant chemotherapy [18]–[20]. Previous studies [21], [22] have reported a detectable increase of diffusion which manifested itself before quantifiable decrease in tumour size, and the diffusion change was observed as early as right upon completion of the first cycle of neoadjuvant chemotherapy. Moreover, preliminary studies have suggested that diffusion may be used as a biomarker for pre-treatment prediction of response to neoadjuvant chemotherapy in patients with locally advanced breast cancer [5], [23], although this hypothesis needs further validation [24]. Diffusion-MRI has also shown potential for evaluating residual breast cancer following neoadjuvant chemotherapy [25].

Several factors, both in data acquisition and processing, can influence the accuracy and precision of quantitative diffusion-MRI measurements [26]–[31]. In particular, it should be noted that the signal-to-noise ratio (SNR) as well as the overall degree of calibration of the high strength diffusion gradients system (which are intrinsically linked to all stages of the diffusion-MRI pipeline, from sequence design through data analysis) can directly and systematically bias the measurement of diffusion indices [32]. Accordingly, some studies have emphasized the importance of implementing specific diffusion-MRI related quality control protocols as well as correction methods [33]–[44], which should be put into practice in addition to standard quality assurance routines in order to guarantee the reliability of quantitative diffusion-MRI measurements. Furthermore, in diffusion-MRI studies, a time- and site-dependency of MR scanner system performance can introduce bias in diffusion-MRI measurements, increase the variance of measured diffusion indices and substantially reduce the power of statistical inference for detecting group differences [30]. In this context, a number of *in vivo* studies have analyzed intra-scanner variability of diffusion-MRI measurements of the brain [27], [28], [45]–[53]. Moreover, given that the integration of multicenter data would greatly improve the sensitivity of diffusion-MRI studies, recent clinical investigations have specifically evaluated the inter-scanner reproducibility of measurements of different diffusion-tensor imaging (DTI)-derived indices in the human brain [54]–[60]. In diffusion-MRI of the body, some *in vivo* studies have evaluated the inter-scan reproducibility of measurements of diffusion indices of the abdomen [61]–[64], liver [65]–[67], prostate [68], anal canal [69] and kidney [70]. However, so far, only a few clinical studies [71]–[74] have specifically investigated the reliability of diffusion-MRI measurements in the breast in terms of inter-scan reproducibility as well as intra- and inter-observer reproducibility.

In view of the fact that breast imaging represents a relatively recent field of application of quantitative diffusion-MRI techniques, and based on the importance of guaranteeing and assessing its reliability in clinical as well as research investigations, the aim of this study was to specifically characterize how the main MR scanner system-related factors affect quantitative measurements in diffusion-MRI of the breast. In particular, we evaluated the accuracy, inter-scan and inter-scanner reproducibility of measurements of phantom diffusion indices performed on 1.5 T MR scanner systems by different manufacturers, all equipped with a dedicated multi-channel breast coil as well as acquisition sequences for diffusion-MRI of the breast.

## Materials and Methods

### 2.1. MR scanner systems and phantom

All diffusion-MRI acquisitions were performed on three commercial 1.5 T MR scanner systems by three different manufacturers, operating in three distinct centers: scanner-A [GE Signa HDx TwinSpeed (GE Medical Systems - Milwaukee, WI, USA) with 50 mT/m maximum gradient strength and 150 T/m/s slew rate], scanner-B [Philips Achieva (Philips Medical Systems - Eindhoven, the Nederlands) with 66 mT/m maximum gradient strength and 90 T/m/s slew rate] and scanner-C [Siemens Avanto (Siemens Healthcare - Erlangen, Germany) with 45 mT/m maximum gradient strength and 200 T/m/s slew rate]. All MR scanner systems were equipped with a dedicated multi-channel breast coil with 8, 7 and 4 elements for scanner-A, scanner-B and scanner-C, respectively. For each MR scanner system, standard maintenance and quality assurance procedures were routinely performed.

The same doped (per 1000 g H_2_O distilled: 1.25 g NiSO_4_ × 6H_2_O + 5 g NaCl) isotropic water phantom (i.e. two identical cylindrical bottles with diameter 11.5 cm and length 20 cm) was employed in all acquisitions.

### 2.2. Data acquisition

Images from different MR scanner systems were obtained using pulse sequences provided by the manufacturers. For diffusion-weighted image acquisition, we used a 2D axial spin echo - echo planar imaging sequence, sensitized to diffusion (DWI-SE-EPI) through strong magnetic field gradient pulses. The acquisition protocols and parameters are reported in Table 1.

**Table 1:** Axial 2D diffusion-weighted spin echo - echo planar imaging (DWI-SE-EPI) sequence: acquisition parameters for scanner-A, scanner-B and scanner-C.

	Scanner-A	Scanner-B	Scanner-C
TR (ms)	8000	8000	8000
TE (ms)	81	81	83
BW (Hz/pixel)	3906	2905	2170
*b-value* (s/mm^2^)	850	850	850
FOV (mm×mm)	350×350	350×350	350×350
Matrix	128×128	128×128	128×128
Slice thickness (mm)	5	5	5
Interslice gap (mm)	0	0	0
Number of slices	21	21	21
K space sampling	5/8	5/8	5/8
Parallel imaging	ASSET	SENSE	mSENSE
Acceleration factor	2	2	2
Phase encoding direction	anterior/posterior	anterior/posterior	anterior/posterior

**Figure 1:**
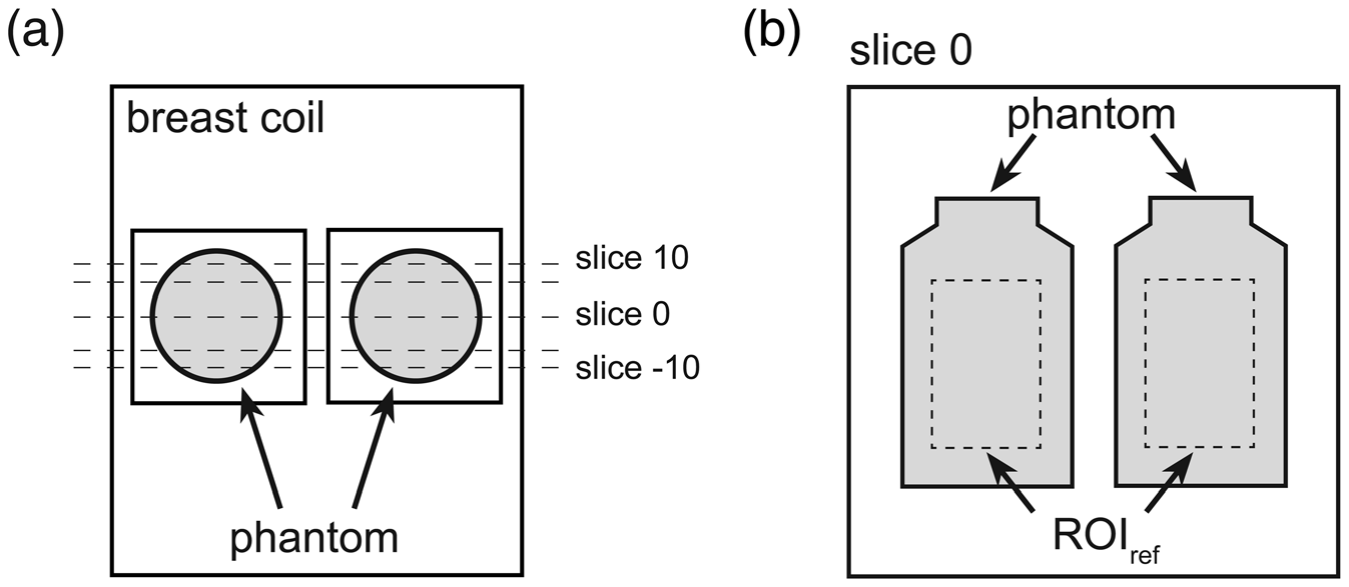
Schematic description of a) breast coil, phantom and acquisition slab (21 slices), as well as b) ROI_ref_ positioning.

For each MR scanner system, all acquisitions were performed on the same day in order to avoid any mid- and long-term changes in scanner performance as well as any potential variability induced by phantom repositioning. The phantom (i.e. two cylindrical bottles as described above) was stored in the scanner room for at least 24 hours prior to scanning and was positioned in the gantry 1 hour before acquisition. The centre of each of the two cylindrical bottles was placed in the centre of each of the two sides of the breast coil and secured using foam padding. The central slice of the acquisition slab (21 slices) was placed at the centre of the two bottles (Figure 1). The temperature of the scanner bore was recorded during data acquisition.

#### 2.2.1. SNR and calibration of diffusion gradients system

In order to evaluate SNR as well as the calibration of the high strength diffusion gradients system in each MR scanner, the axial DWI-SE-EPI sequence (Table 1) was acquired both without (*b-value* = 0 s/mm^2^) and with (*b-value* = 850 s/mm^2^) sensitization to diffusion along each of the main orthogonal directions (readout/left-right, phase-encoding/anterior-posterior, slice-selection/head-foot). In order to improve SNR, we employed a number of excitations (NEX) equal to 14. The above acquisitions were repeated obtaining a total of 5 measurements.

#### 2.2.2. Accuracy, inter-scan and inter-scanner reproducibility of diffusion-MRI measurements

In order to assess the accuracy, inter-scan and inter-scanner reproducibility of measurements of conventional mean apparent diffusion coefficient (ADC) measured along the main orthogonal directions (<ADC>) as well as DTI-derived indices (which a few breast studies have preliminarily incorporated in their scanning protocols [72], [73], [75]–[77]) additional diffusion-weighted images along each of the main orthogonal directions and DTI data sets were acquired using the DWI-SE-EPI sequence (Table 1) with *b-value* = 850 s/mm^2^. For each MR scanner system, DTI acquisitions of the DWI-SE-EPI sequence with sensitization to diffusion along 6 non-collinear and non-coplanar directions [72], [75], [76] were performed. For <ADC> as well as DTI measurements, an additional acquisition of the DWI-SE-EPI sequence without sensitization to diffusion (*b-value* = 0 s/mm^2^) was carried out. In order to guarantee a constant acquisition time, the NEX value was 7 and 4 for <ADC> and DTI data sets, respectively. The entire set of acquisitions was repeated obtaining a total of 5 measurements for both <ADC> and DTI data.

### 2.3. Image processing and analysis

Except for diffusion tensor estimation, all processing and analysis of diffusion-MRI data was performed using custom scripts developed in MATLAB 7.1 (MathWorks, Natick, MA, USA).

In order to better evaluate inter-scanner variability of diffusion-MRI measurements independently of temperature (T_a_) during data acquisition, ADC measured along each of the main orthogonal directions, <ADC> and DTI-derived mean diffusivity (MD) values were normalized to a reference value corresponding to a temperature of 22°C (at which the phantom diffusion coefficient, D_0_, is equal to 2.14±0.03×10^−3^ mm^2^/s) [78]. In particular, for each MR scanner system, we used an analytical equation derived by fitting experimental water diffusion coefficients measured at different temperature values with the Arrhenius activation law to obtain the true phantom diffusion coefficient at T_a_ (D_a_) [78]. Given that for an isotropic phantom the ratio (R) between the value of a diffusion index measured at T_a_ and D_a_ depends exclusively on the ratios (R_b_^i = 1-n^, n = 1, 3 and 6 for ADC measured along each of the main orthogonal directions, <ADC> and MD, respectively) between the nominal and the effective *b-value* applied along the diffusion sensitized directions (which can reasonably be considered independent of temperature), the normalized values of diffusion indices were calculated as RD_0_.

All analyses were carried out in the central slice of the acquired phantom volume, within a reference region of interest (ROI_ref_) that consisted of two rectangles (size 29×41 voxels), each placed in the centre of the image of the bottle on each of two sides of the breast coil (Figure 1).

#### 2.3.1. SNR and maps of ADC along each of the main orthogonal directions

The SNR was calculated using non-diffusion-weighted (b_0_) images. Conventional approaches to evaluate SNR are based on the signal statistics in one or two separate large regions of interest of a single image or the signal statistics in a large region of interest of a difference image of two repeated acquisitions [79], [80]. In order to take into account spatial variations in SNR (which can be substantial in acquisitions performed using multi-channel coils and parallel imaging techniques) [81], maps of SNR in small adjacent ROIs of 8×8 voxels (SNR_ROI_) were computed as previously described [81], [82]:
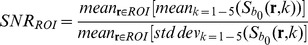
(1)where S_b0_(**r**,k) is the signal of the voxel at position **r** within the selected ROI for the *k*th repetition of the b_0_ image. The overall SNR was computed as the mean value of SNR_ROI_ within ROI_ref_.

For each repetition (*k = *1–5), ADC maps along each of the main orthogonal directions [ADC_i,k_(**r**) **-** *i* = 1, readout/left-right; *i* = 2, phase-encoding/anterior-posterior; *i* = 3, slice-selection/head-foot] were computed. For the *i*th direction, the mean, (ADC_i_)_mean_, and standard deviation, (ADC_i_)_SD_, images across repetitions were calculated. Then, the overall ADC along the *i*th diffusion weighting direction (ADC_i_) was obtained as the average (ADC_i_)_mean_ within ROI_ref_. Furthermore, the overall percent coefficient of variation for repeated measurements of ADC along the *i*th diffusion weighting direction was computed as follows:
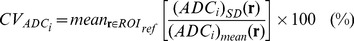
(2)


The spatial non-uniformity levels of maps of ADC along each of the main orthogonal directions were evaluated by adapting a method proposed by Magnusson and Olsson [83]. The ADC_i,k_ maps were smoothed using a low-pass spatial filter with a 3×3 kernel which reduces noise by computing the mean value of a voxel and its 8 neighbours, and replacing the value of the voxel with this mean. Then, the mean value (C) within ROI_ref_ was estimated. For each voxel, the deviation from this value was calculated as the absolute value of [100×(voxel value - C)/C], obtaining a new image which represents the absolute value of the percentage deviation from C. The mean value of this new image within ROI_ref_ was recorded, obtaining the non-uniformity value of ADC_i,k_ maps (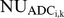) for each diffusion weighting direction (*i = *1–3) and repetition (*k = *1–5). Finally, for each diffusion weighting direction, the overall non-uniformity degree (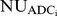) was estimated as the mean of 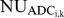 across repetitions.

#### 2.3.2. Maps of <ADC>, MD and FA

For each repetition (*k = *1–5), the mean ADC along the main orthogonal directions [<ADC>_k_(**r**)] was calculated voxel-wise. The overall mean ADC (<ADC>) and its coefficient of variation for repeated measurements (CV_<ADC>_) were calculated as described above for ADC_i_ and 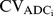, respectively. Moreover, the overall spatial non-uniformity degree of <ADC> maps (NU_<ADC>_) was estimated using the same method employed for calculating 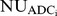.

In order to estimate the diffusion tensor, we adopted a method similar to that described in previous breast DTI studies [72], [76]. In particular, we performed the standard steps implemented in the diffusion toolbox (FDT) of FSL 4.1.4 (Oxford Centre for Functional Magnetic Resonance Imaging of the Brain (FMRIB) software library) [84], [85] using the weighted linear least square approach. For each repetition (*k = *1–5), the mean diffusivity [MD_k_(**r**)] and fractional anisotropy [FA_k_(**r**)] were computed voxel-wise. Then, the overall mean diffusivity (MD) and fractional anisotropy (FA) were calculated as described for ADC_i_. The coefficient of variation for repeated measurements of MD (CV_MD_) and spatial non-uniformity of MD maps (NU_MD_) were then obtained using the same procedure adopted for 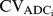 and 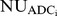
calculation.

#### 2.3.3. Statistical analysis

Any significant difference in quality control data and measured diffusion metrics, both across the main orthogonal directions within a single MR scanner system and across MR scanner systems, was assessed through a one-way analysis of variance (ANOVA). When the ANOVA revealed a significant difference (p<0.05), a post-hoc analysis was performed using the two sample t-test, with Bonferroni correction for multiple comparisons. For each MR scanner system, any significant difference between <ADC> and MD maps was assessed similarly. The one-sample t-test, with Bonferroni correction for multiple comparisons, was used to evaluate any significant difference between the true diffusion indices and estimated diffusion indices.

## Results

### 3.1. SNR and calibration of diffusion gradients system

The SNR (mean value ± standard deviation within ROI_ref_) was 242±55, 184±26 and 309±60 for scanner-A, scanner-B and scanner-C, respectively. The SNR values varied significantly across MR scanner systems (ANOVA: p<0.0001 – post-hoc analysis: p<0.001 for scanner-A vs scanner-B, scanner-A vs scanner-C and scanner-B vs scanner-C).

**Figure 2:**
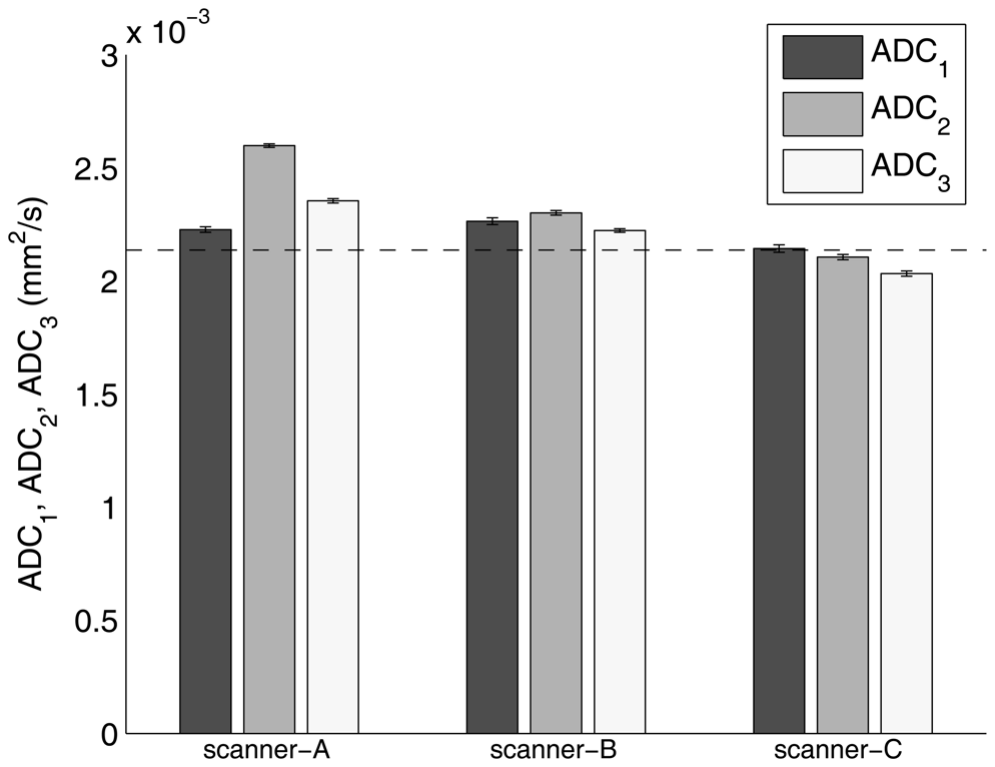
Phantom ADC along each of the main orthogonal directions (ADC_i_ - *i* = 1, readout/left-right; *i* = 2, phase-encoding/anterior-posterior; *i* = 3, slice-selection/head-foot) for scanner-A, scanner-B and scanner-C. The bar charts depict the mean of the average value within ROI_ref_ ± standard deviation across five repetitions. The dashed line represents the known phantom diffusion coefficient (2.14±0.03×10^−3^ mm^2^/s) at the reference temperature value of 22°C.

**Figure 3:**
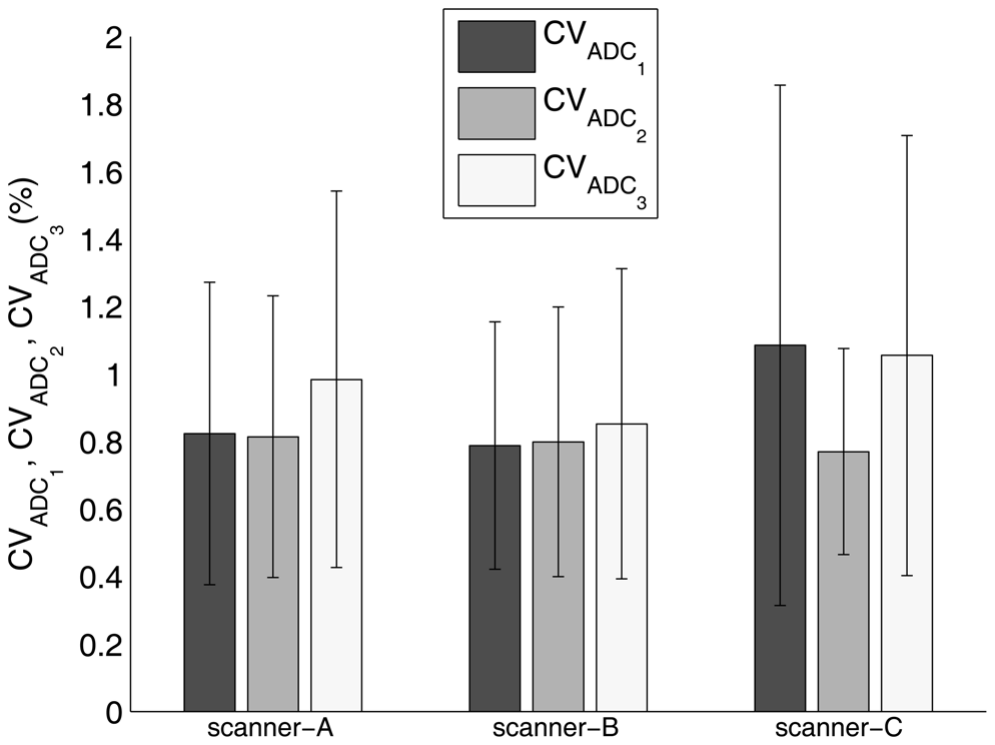
Coefficient of variation for repeated measurements of phantom ADC along each of the main orthogonal directions (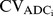 - *i* = 1, readout/left-right; *i* = 2, phase-encoding/anterior-posterior; *i* = 3, slice-selection/head-foot) for scanner-A, scanner-B and scanner-C. The bar charts depict the mean value ± standard deviation within ROI_ref_.

**Figure 4:**
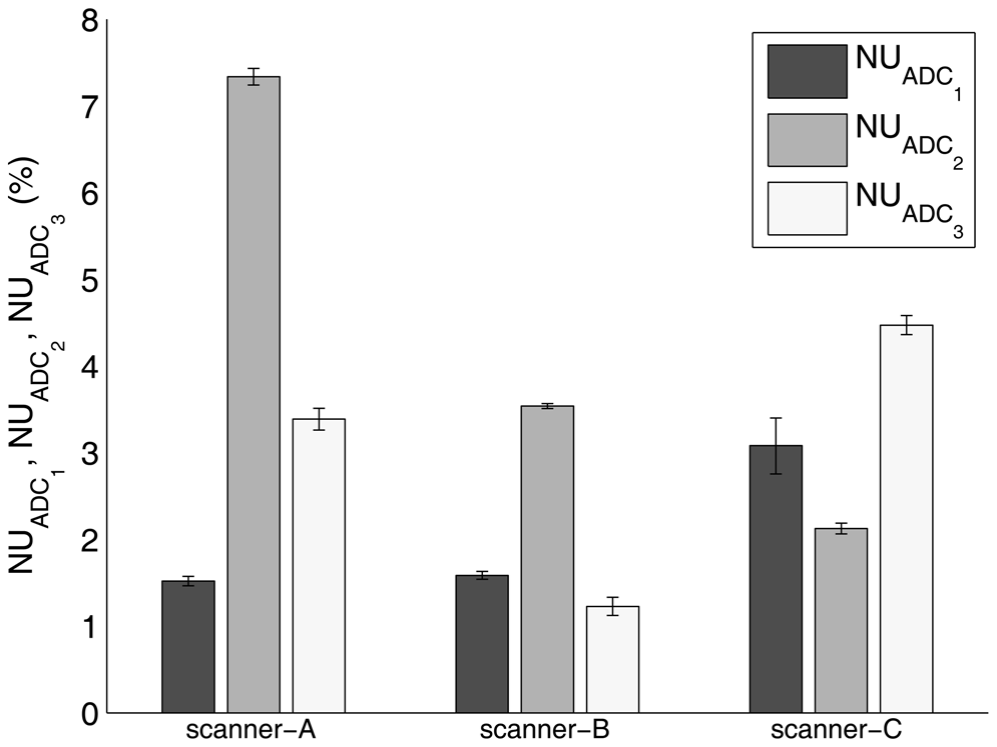
Non-uniformity of maps of phantom ADC along each of the main orthogonal directions (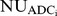 - *i* = 1, readout/left-right; *i* = 2, phase-encoding/anterior-posterior; *i* = 3, slice-selection/head-foot) for scanner-A, scanner-B and scanner-C. The bar charts depict the mean value ± standard deviation across five repetitions.

For each of the main orthogonal directions (*i* = 1–3), the ADC_i_, 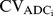 and 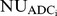 results are reported in Figure 2, Figure 3 and Figure 4, respectively. ADC_i_ (*i* = 1–3) values varied significantly with diffusion gradient direction for scanner-A (ANOVA: p<0.0001 – post-hoc analysis: p<0.001 for *i* = 1 vs *i* = 2, *i* = 1 vs *i* = 3, *i* = 2 vs *i* = 3), scanner-B (ANOVA: p<0.0001 – post-hoc analysis: p<0.001 for *i* = 1 vs *i* = 2, *i* = 1 vs *i* = 3, *i* = 2 vs *i* = 3) and scanner-C (ANOVA: p<0.0001 – post-hoc analysis: p<0.01 and p<0.001 for *i* = 1 vs *i* = 2 and *i* = 1 vs *i* = 3, *i* = 2 vs *i* = 3, respectively). Moreover, except for ADC_1_ for scanner-C (p>0.05), all measured ADC_i_ values were significantly (p<0.05) different from the known phantom diffusion coefficient. For each MR scanner system, 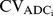 and 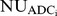 values varied significantly with diffusion gradient direction (ANOVA: p<0.0001 – post-hoc analysis: p<0.001 for *i* = 1 vs *i* = 2, *i* = 1 vs *i* = 3, *i* = 2 vs *i* = 3).

### 3.2. Accuracy, inter-scan and inter-scanner reproducibility of diffusion-MRI measurements

**Figure 5:**
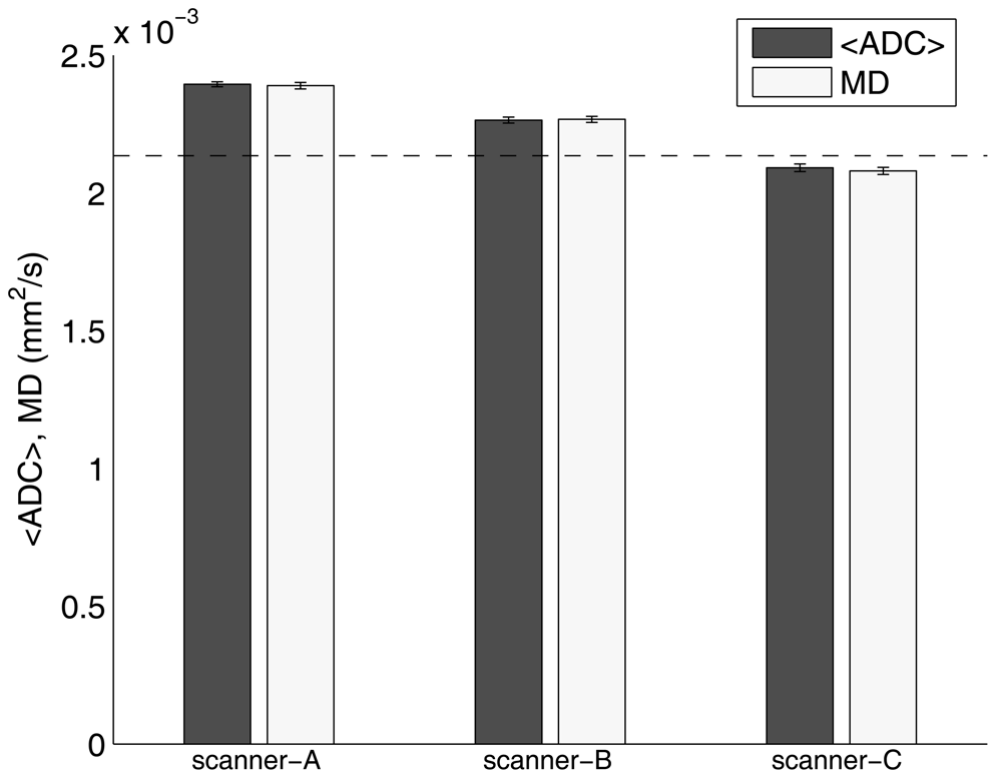
Mean ADC along the main orthogonal directions (<ADC>) as well as mean diffusivity (MD) of the phantom for scanner-A, scanner-B and scanner-C. The bar charts depict the mean of the average value within ROI_ref_ ± standard deviation across five repetitions. The dashed line represents the known phantom diffusion coefficient (2.14±0.03×10^−3^ mm^2^/s) at the reference temperature value of 22°C.

Both <ADC> and MD data are reported in Figure 5. Within each MR scanner system, <ADC> and MD values were not significantly different (p>0.05) from each other. On the other hand, <ADC> and MD values varied significantly across MR scanner systems (ANOVA: p<0.0001 – post-hoc analysis: p<0.001 for scanner-A vs scanner-B, scanner-A vs scanner-C and scanner-B vs scanner-C). Both <ADC> and MD values were significantly (p<0.01) different from their true value, with an accuracy (percentage difference between the measured and known diffusion value) ranging from -2.6% to 12.0%.

The CV_<ADC>_ and CV_MD_ results as well as NU_<ADC>_ and NU_MD_ results are reported in Table 2. For each MR scanner system, CV_<ADC>_ and CV_MD_ differed significantly (p<0.001) with values which were less than 1%. Moreover, CV_<ADC>_ and CV_MD_ varied significantly across MR scanner systems (ANOVA: p<0.0001 – post-hoc analysis: p<0.001 for scanner-A vs scanner-B, scanner-A vs scanner-C and scanner-B vs scanner-C). For scanner-A and scanner-B, NU_<ADC>_ and NU_MD_ were not significantly (p>0.05) different, whereas, for scanner-C, NU_<ADC>_ was significantly (p<0.01) lower than NU_MD_. Furthermore, both NU_<ADC>_ (ANOVA: p<0.0001 – post-hoc analysis: p<0.001 and p>0.05 for scanner-A vs scanner-B, scanner-A vs scanner-C and scanner-B vs scanner-C, respectively) and NU_MD_ (ANOVA: p<0.0001 – post-hoc analysis: p<0.001 for scanner-A vs scanner-B, scanner-A vs scanner-C and scanner-B vs scanner-C) varied significantly across MR scanner systems, with values which were less than 4%.

**Table 2:** CV_<ADC>_ and CV_MD_ results [mean value (standard deviation within ROI_ref_)] as well as NU_<ADC>_ and NU_MD_ results [mean value (standard deviation across five repetitions)] for scanner-A, scanner-B and scanner-C.

	Scanner-A	Scanner-B	Scanner-C
CV_<ADC>_ (%)	0.69 (0.34)	0.64 (0.31)	0.81 (0.38)
CV_MD_ (%)	0.53 (0.28)	0.67 (0.32)	0.92 (0.58)
NU_<ADC>_ (%)	3.70 (0.04)	1.70 (0.08)	1.83 (0.14)
NU_MD_ (%)	3.65 (0.09)	1.67 (0.08)	2.52 (0.09)

For each MR scanner system, the overall FA of the phantom was significantly (p<0.001) greater than 0. Moreover, FA values varied significantly across MR scanner systems (ANOVA: p<0.0001 – post-hoc analysis: p<0.001 for scanner-A vs scanner-B, scanner-A vs scanner-C and scanner-B vs scanner-C). In particular, FA values (mean of the average value within ROI_ref_ ± standard deviation across five repetitions) were 0.086±0.001, 0.050±0.001 and 0.076±0.001 for scanner-A, scanner-B and scanner-C, respectively.

## Discussion

A number of *in vivo* studies have evaluated the reliability of diffusion-MRI measurements in the brain as well as body [27], [28], [45]–[70], [86]. However, a more specific and careful evaluation of the reliability of diffusion-MRI measurements of the breast would be of practical interest. Recently, O'Flynn *et al.* [71] have assessed the mid-term reproducibility and inter-observer variability of ADC measurements of fibroglandular tissue at 3 T, obtaining a within-subject coefficient of variation of 22–25% and a kappa value (κ) of 0.83. Partridge *et al.* [72] have evaluated the reproducibility of DTI-derived parameter measurements in normal breast tissue at 1.5 T after repositioning and rescanning, reporting a between-scan coefficient of variation of 4.5% and 11.4% for MD and FA, respectively. Tagliafico *et al.* [73] have reported a between-scan coefficient of variation of 15% and 30% for MD and FA measurements in normal breast tissue at 3 T, respectively; moreover, when looking at intra-/inter-observer variability, the κ values were 0.82–0.89/0.73–0.83 and 0.60–0.84/0.64–0.80 for MD and FA, respectively. Additionally, when measuring ADC at 1.5 T in breast carcinomas, Petralia *et al.* [74] estimated an intra- and inter-observer variability of 1.1% and 2%, respectively. It should be noted that assessing and guaranteeing reliability of quantitative diffusion-MRI measurements, which is a prerequisite for successful clinical as well as research studies, necessarily includes a characterization of the MR scanner system. Indeed, although *in vivo* studies can evaluate repeatability and reproducibility of diffusion-MRI measurements in a clinical setting (which are fundamental elements toward carrying out longitudinal as well as multicenter studies), such studies do not allow to address measurement accuracy as well as some of the main characteristics of MR scanner system (e.g. the SNR or the calibration of high strength diffusion gradients system) that can systematically bias quantitative diffusion-MRI measurements [30], [32]. A limited number of studies have reported phantom data specific to the characterization of MR scanner systems for diffusion-MRI of the brain as well as the body [34]–[41], [43], [57], [58], [87], [88]. However, in diffusion-MRI of the breast, only a few clinical studies have incorporated a basic verification of the calibration of diffusion gradients [11], [22], [72], [89], [90].

To the best of our knowledge, this is the first phantom study which carries out multiple and specific quality controls in order to characterize in detail different 1.5 T MR scanner systems by three different manufacturers, all equipped with a dedicated multi-channel breast coil as well as acquisition sequence for quantitative diffusion-MRI of the breast. In particular, for each MR scanner system, we evaluated the calibration of high strength diffusion gradients for the three main orthogonal axes along which diffusion-sensitizing gradients can be applied. Then, we assessed how the MR scanner system-related factors affect the accuracy, inter-scan and inter-scanner reproducibility of diffusion measurements of <ADC> as well as of DTI measurements of MD. We used acquisition protocols and parameters typically employed in diffusion-MRI of the breast, which, except for small differences in readout bandwidth (BW) values, were similar for all MR scanner systems. As suggested by Bogner *et al.* [89], we employed a *b-value* of 850 s/mm^2^. For all acquisitions, we used the same homogeneous and isotropic phantom with known diffusion coefficient, allowing a proper evaluation of the accuracy of estimated diffusion indices as well as non-uniformity of maps of diffusion indices. The diffusion coefficient of the phantom at room temperature (∼2×10^−3^ mm^2^/s) is similar to water diffusion coefficient in normal breast tissue (1.8–2.1×10^−3^ mm^2^/s), while it is slightly higher than water diffusion coefficient in malign as well benign breast tissue (0.9–1.7×10^−3^ mm^2^/s) [7], [22], [72], [89], [91]. In this context, as previously described by Delakis *et al.* [35], the use of a phantom with a relatively high diffusion coefficient is recommended in order to improve the sensitivity to any discrepancies in measured diffusion indices induced by differences between the nominal and the effective *b-value* applied along the diffusion sensitized directions.

Diffusion-MRI measurements are affected by an inherently low SNR. In particular, both precision and accuracy of diffusion indices can depend on SNR [27]–[29], [82], [92]–[94]. Therefore, we began by characterizing each MR scanner system in terms of SNR. In particular, the overall SNR of scanner-B was 24% and 40% lower than that of scanner-A and scanner-C, respectively. Interestingly, based on the BW values of the acquisition protocols (Table 1), scanner-A (highest BW value) was expected to show the lowest SNR across MR scanner systems. Therefore, SNR results cannot be ascribed to differences in BW values only, and are likely to also reflect different overall sensitivities of the breast coils.

All MR scanner systems showed a high short term stability of the performance of diffusion gradients. For each MR scanner system, the overall coefficient of variation for repeated measurements of ADC along each of the main orthogonal directions was less than 1.1% (Figure 3). Nonetheless, for each MR scanner system and direction (readout/left-right, phase-encoding/anterior-posterior, slice-selection/head-foot) except for ADC measurements along the readout/left-right direction for scanner-C, we revealed a significant difference between the measured ADC and the true diffusion coefficient. Moreover, for each MR scanner system, the entity of this difference varied significantly with diffusion direction (Figure 2). This effect, when quantified in terms of the coefficient of variation of ADC measurements across the main orthogonal directions, was more relevant for scanner-A (7.9%) as compared to scanner-B (1.7%) and scanner-C (2.7%). As a whole, these results indicate a mismatch between the theoretically assumed and the effective *b-value*. This could originate from errors in diffusion gradients amplitude, eddy current fields, concomitant field terms and cross terms between diffusion gradients and imaging gradients [2], [32], [87], [88], [95]. These factors are direction-dependent and can have deleterious effects that are more prominent at the high gradient strengths usually employed in diffusion-MRI [2], [32]. In addition, any diffusion gradient non-uniformity is expected to yield a spatial variation in measured diffusion indices. For each MR scanner system, we observed that the spatial non-uniformity values of maps of ADC along each of the main orthogonal directions depended significantly on the diffusion weighting direction. Scanner-A showed a relatively high spatial non-uniformity value (7.3%) of ADC along the phase-encoding/anterior-posterior direction, while for both scanner-B and scanner-C the degree of non-uniformity of ADC along each diffusion weighting direction was less than 4.5% (Figure 4). In general, when DWI-SE-EPI sequences (Table 1) are acquired, the high strength diffusion gradients system belonging to each MR scanner system presented an overall mis-calibration (not documented by standard maintenance procedures and quality assurance routines), which can affect diffusion indices measurement. Therefore, in order to improve the reliability of quantitative diffusion-MRI of the breast, suitable correction methods could be employed [34], [36], [37], [40].

For each MR scanner system, the coefficient of variation for short term repeated measurements of both <ADC> and MD was less than 1% (Table 2), while previous clinical studies [71]–[73] which measured breast diffusion indices have reported a between-scan coefficient of variation in the range 5–15%. The greater experimental variability of *in vivo* diffusion-MRI measurements when compared to our phantom study is likely due to patient repositioning, manual ROI positioning and motion induced effects.

For every MR scanner system, the spatial non-uniformities of <ADC> and MD maps were less than 4% (Table 2). For scanner-A and scanner-B, non-uniformities of <ADC> and MD maps were not significantly different. Conversely, for scanner-C, the non-uniformity of the <ADC> map (1.8%) was significantly lower than the non-uniformity of the MD map (2.5%). For each MR scanner system, we did not reveal any significant difference between estimated <ADC> and MD values, with an absolute percentage difference between <ADC> and MD of less than 0.6%. This indicates a correct pulse timing when using multiple oblique diffusion gradients as employed in DTI, and may suggest the negligibility of cross-term effects between diffusion and imaging gradients along different directions [26], [95], [96]. However, the accuracy of <ADC> and MD measurements varied significantly with the MR scanner system (Figure 5). In particular, the mean value of <ADC> and MD accuracies was 11.9%, 6.0% and -2.3% for scanner-A, scanner-B and scanner-C, respectively, while the mean value of the coefficients of variation for <ADC> and MD measurements across MR scanner systems was 6.8%. Previous phantom studies of diffusion-MRI both using a head coil [35], [36], [38]–[40], [43], [58], [87] and a body coil [34], [43] have reported accuracy values of estimated diffusion indices ranging from −15% to 30%. Other *in vivo* studies of the brain [45], [46], [54]–[59] have reported a coefficient of variation in MD and FA across different MR scanner systems in the ranges 4–15% and 5–29%, respectively. In this context, it is important to note that the differences in diffusion indices reported in previous clinical diffusion-MRI studies of the breast range from 5% to 45% [9]–[13], [17], [21]–[23], [25], [76]. Therefore, a comparison of breast diffusion-MRI data from different centers should be performed with great caution. Moreover, during the planning of a multicenter study, the accuracy of diffusion-MRI measurements should be carefully assessed in every participating center. Additionally, in longitudinal studies, a periodic monitoring of the accuracy of measured diffusion indices is highly recommended. In a meta-analysis of 13 studies dealing with quantitative diffusion-MRI in the differential diagnosis of breast lesions, Chen *et al*. [91] have shown that a) the ADC values of benign lesions ranged from 1×10^−3^ mm^2^/s to 1.82×10^−3^ mm^2^/s, b) the cutoff values for differentiating malignant from benign lesions ranged from 0.9×10^−3^ mm^2^/s to 1.76×10^−3^ mm^2^/s, and that c) the sensitivity and specificity ranged from 63% to 100% and 46% to 97%, respectively. This heterogeneity could be due to differences in patient characteristics and diagnostic criteria, as well as to different diffusion-MRI acquisition and analysis methods. However, we hypothesize that potential differences in MR scanner system-related factors between different MR scanner systems, which can systematically bias accuracy and precision of diffusion-MRI measurements, may contribute to explaining the results heterogeneity reported by Chen *et al.* [91].

Besides DTI-based measurements of MD, we also performed diffusion anisotropy estimation, and the overall FA value of the isotropic phantom (true FA = 0) was found to be significantly greater than 0 for every MR scanner system. This could reflect effects of relatively low SNR (high SNR has been shown to reduce the brain anisotropy overestimation due to noise at a *b-value* typically used in clinical DTI examinations, ∼1000 s/mm^2^) [93], [94], as well as errors in diffusion gradients amplitude (which can result in mimicking anisotropy). While for each MR scanner system FA values were less than 0.09, they were significantly different among MR scanner systems. In particular, data acquired on scanner-B resulted in the lowest FA estimate (∼0.05).

## Conclusions

Although breast imaging is an appealing and promising application field of diffusion-MRI, only few *in vivo* studies have recently evaluated the inter-scan reproducibility as well as intra- and inter-observer reproducibility of diffusion measurements of the breast [71]–[74]. In this phantom study, we characterized in detail three 1.5 T MR scanner systems by three different manufacturers, all equipped with a dedicated multi-channel breast coil as well as acquisition sequences for quantitative diffusion-MRI of the breast. The SNR as well as overall calibration of high strength diffusion gradients system varied substantially across MR scanner systems, introducing systematic bias in measurements of diffusion indices. We note that *in vivo* diffusion-MRI measurements of the breast can also depend on other non-MR scanner system-related factors – such as subject-related artifacts (e.g. motion and cardiac pulsation, physiological noise), perfusion and non-Gaussian processes – that could further increase the variability in diffusion measurements. Nonetheless, in order to improve the reliability of quantitative breast diffusion-MRI and, hence, the sensitivity of clinical studies, a specific and periodic quality control program for characterizing and monitoring the performance of breast coil and high strength diffusion gradients of MR scanner system is highly recommended at every site, especially before multicenter studies are tackled as well as during longitudinal studies. In this context, we agree with Jones [30] and De Santis *et al.* [41] who have recently emphasized that the quality control culture in diffusion-MRI remains limited. Therefore, we feel that in diffusion-MRI, which is a truly quantitative technique, enabling a suitable and dedicated quality assurance program at every site would represent a major step toward the effective use of every MR scanner system as a “measurement tool”, hence further improving and strengthening the capabilities of this powerful diagnostic modality.

## Supporting Information

Figure S1 **Maps of phantom ADC along each of the main orthogonal directions (** ***i*** **= 1, readout/left-right;** ***i*** **= 2, phase-encoding/anterior-posterior;** ***i*** **= 3, slice-selection/head-foot), calculated using the first (** ***k*** **= 1) of 5 repetitions (ADC_i,1_), for scanner-A (left pane), scanner-B (middle pane) and scanner-C (right pane).** In order to facilitate visual assessment, the figure depicts a zoomed region (located on one side of the breast coil) of the phantom containing one of the two rectangular ROIs (highlighted in red) which make up ROI_ref_.
(TIF)
