# Supplementary figures and images for: MR Scanner Systems Should Be Adequately Characterized in Diffusion-MRI of the Breast
Source: PLoS One. 2014 Jan 28;9(1):e86280. doi: 10.1371/journal.pone.0086280 (PMC3904912; doi:10.1371/journal.pone.0086280)

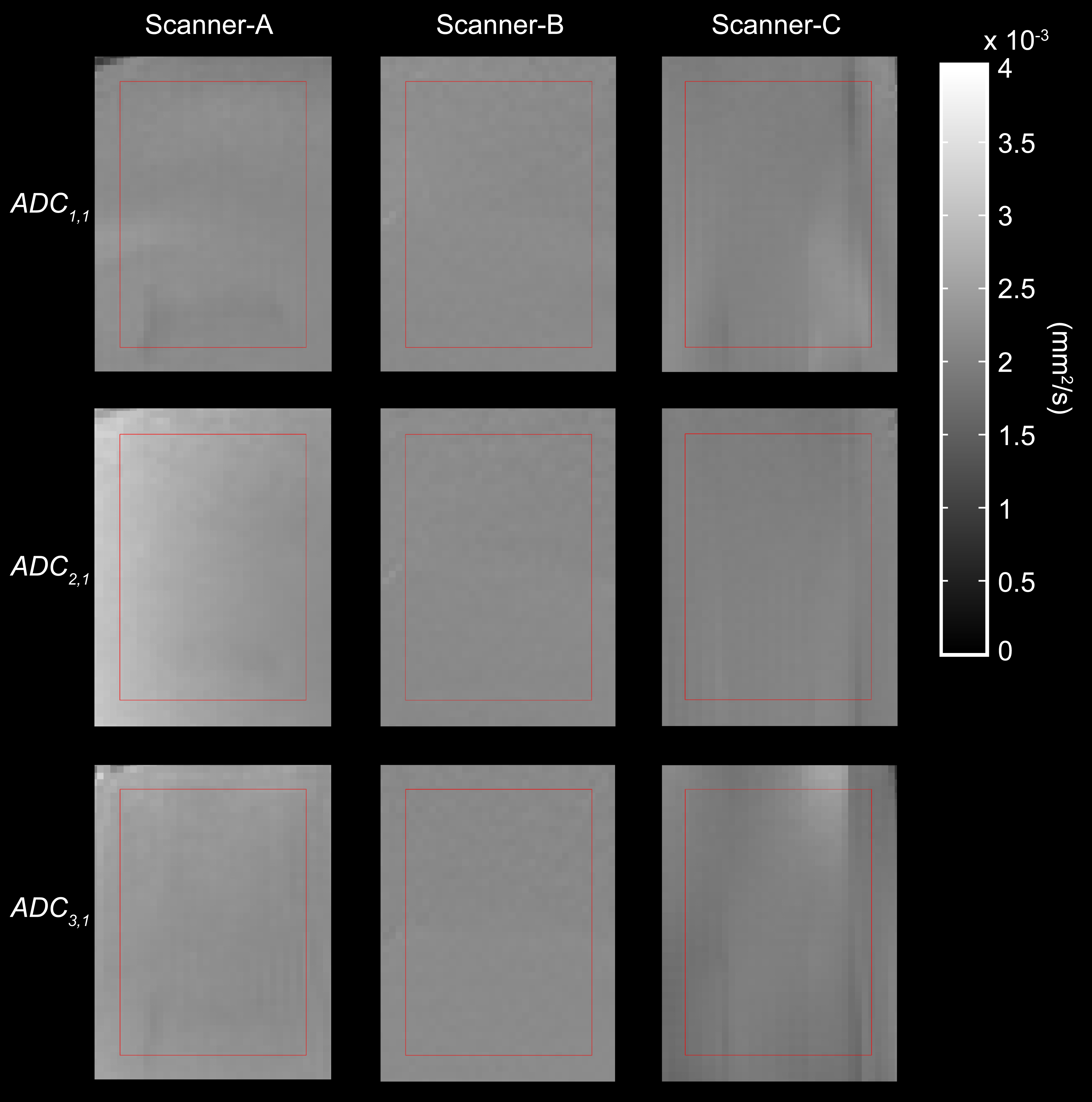

Supplement: Figure S1 — Maps of phantom ADC along each of the main orthogonal directions ( i = 1, readout/left-right; i = 2, phase-encoding/anterior-posterior; i = 3, slice-selection/head-foot), calculated using the first ( k = 1) of 5 repetitions (ADCi,1), for scanner-A (left pane), scanner-B (middle pane) and scanner-C (right pane). In order to facilitate visual assessment, the figure depicts a zoomed region (located on one side of the breast coil) of the phantom containing one of the two rectangular ROIs (highlighted in red) which make up ROIref. (TIF) [file pone.0086280.s001.tif]
